# Effect of Probiotics on Oral Candidiasis: A Systematic Review and Meta-Analysis

**DOI:** 10.3390/nu11102449

**Published:** 2019-10-14

**Authors:** Tiziana Mundula, Federica Ricci, Beatrice Barbetta, Michela Baccini, Amedeo Amedei

**Affiliations:** 1 Unit of Biostatistics, Epidemiology and Public Health. University of Padova, 35131 Padova, Italy; greentea@virgilio.it; 2 Department of Experimental and Clinical Medicine, University of Florence, 50134 Florence, Italy; federica.ricci1987@gmail.com; 3 Department of Biostatistics, Rottapharm Biotech, 20900 Monza, Italy; beatrice.barbetta@rottapharmbiotech.com; 4 Department of Statistics, Computer Science, Applications, University of Florence, 50134 Florence, Italy; 5 SOD of Interdisciplinary Internal Medicine, Azienda Ospedaliera Universitaria Careggi (AOUC), 50134 Florence, Italy

**Keywords:** *Candida* spp., oral candidiasis, *Candida* spp. treatment, *Candida* spp. prevention, *Candida* spp. carriage, probiotics, microbiota, Bayesian meta-analysis

## Abstract

Oral candidiasis (OC) is an increasing health problem due to the introduction of new drugs, population aging, and increasing prevalence of chronic illness. This study systematically reviews the effects of the oral intake of probiotics, prebiotics, and synbiotics on *Candida* spp. counts (colony-forming units (CFU)/mL) in oral and palatal samples. A literature search was conducted. Twelve studies, eight randomized clinical trials (RCTs), and four pre-post studies, resulted as eligible for the meta-analysis, which was performed through a Bayesian random-effects model. All studies analyzed probiotics, and none of them analyzed prebiotics or synbiotics. The treatments effects were measured in terms of odds ratio (OR) of OC (CFU/mL >10^2^, 10^3^, or 10^4^). The meta-analytic OR was 0.71 (95% credibility interval (CrI): 0.37, 1.32), indicating a beneficial effect of treatment; the *I^2^* index was 56.3%. Focusing only on RCTs, the OR was larger and more precise at 0.53 (95% CrI: 0.27, 0.93). The effect of treatment appeared to be larger on denture wearers. Our findings indicate that the intake of probiotics can have a beneficial effect on OC and that the effects could vary according to the patients’ characteristics. Due to the presence of medium–high-risk studies, the results should be interpreted with caution.

## 1. Introduction

*Candida* spp. represent a commensal yeast belonging to the normal microbiota localized on the surface of different body sites (skin, oral cavity, and the gastro-intestinal, uro-genital, and respiratory tracts) of human beings [1]. *Candida* spp. colonization of the mucus membranes occurs very early in life, usually at birth [2]. Under specific conditions, the fungus can switch from a harmless form into a pathogenic form that can lead to infections [3]. About 75% of healthy adults carry *Candida* spp. in the mouth; when there is a detection of a salivary *Candida* spp. count >400 colony-forming units (CFU) per mL, an infection occurs called “oral candidiasis” (OC) [4]. OC is predominantly caused by *Candida albicans* and by other species like *Candida parapsilosis*, *Candida metapsilosis, Candida tropicalis*, *Candida khmerensis* [5], *Candida glabrata* [6], and *Candida dubliniensis* [7]. Using a clinical evaluation, we can identify different *Candida* spp. infection phenotypes: pseudomembranous, erythematous, hyperplastic, angular cheilitis, median rhomboid glossitis [8,9], denture stomatitis [10], and linear gingival erythema [11]. All these conditions can determine a widespread spectrum of symptoms ranging from asymptomatic to very severe (such as burning sensation, pain, lesions, and bleeding), leading to discomfort in mastication, thereby limiting the food intake.

OC incidence is growing in the last few decades, because of the increase in some immune-correlated chronic illnesses (diabetes, cancer, human immunodeficiency virus (HIV)) and the intensive use of some drugs, such as antibiotics, chemotherapy, and immunosuppressants [12]. Some of major factors contributing to OC development are summarized in Table 1.

Sometimes, the superficial infection can spread out into the body, into the blood stream, causing deep and invasive candidiasis, which is associated with high hospitalization rate and even mortality [13]. The available pharmacological treatments (e.g., antifungal drugs) are very effective but present some critical points, such as frequent side effects and, in particular, antifungal resistance [1]. Therefore, it would appear critical to develop new prophylactic and complementary therapeutic strategies. The intake of probiotics seems a promising method in order to achieve these purposes. In fact, they can modulate the gut microbiota and its cross-talk with immune response, with local (intestinal) and systemic relapses [25,26,27,28,29].

Probiotics, that were identified and studied at the end of 19th century by various scientists such as Metchnikoff, Tissier, Grigorov, and Shirota, are defined as “live microorganisms that, when administered in adequate amount, confer health benefit to the host” [30]. The most used probiotics belong to *Lactobacillus* spp. and *Bifidobacterium* spp. and, to a lesser extent, to *Saccharomyces* spp., *Bacillus* spp., and *Escherichia* spp. [31]. The beneficial proprieties of probiotics are supported by various in vitro and in vivo studies, which used different bacterial strains (single or in combination), at different dosages [31,32]. Various studies proved the preventive and therapeutic effects of good bacteria, some of which involve metabolic functions such as fermentation of indigestible fibers [33], short-chain fatty-acid production [34], lactose tolerance [35], vitamin production [36], and reduction of cholesterol levels [37]. In addition, good bacteria have antimicrobial activity (such as competitive inhibition of pathogens [38]), produce bacteriocins [39], have antitoxin effects [40], and enhance the intestinal barrier function [41] (e.g., increased production of mucins, tight junction proteins, and goblet and Paneth cells [42]). Finally, commensal bacteria exercise immune modulation (such as the stimulation of immunoglobulin A (IgA) production, increased production of anti-inflammatory cytokines, and induction of regulatory T cells [42]).

These probiotics’ proprieties suggested their use for the treatment and prevention of many medical conditions (diarrhea, constipation, inflammatory bowel disease, irritable bowel syndrome, allergic disease), sometimes with excellent results [31]. In addition, probiotics also showed an antifungal action and were successfully used in mucosal candidiasis, as reported in an in vivo study by Wagner in 1997 [43].

Sookkhee et al., in 2001, studied the effects on *Candida albicans* growth of different lactic-acid bacteria isolated from the oral cavity of volunteers and found that two strains, *Lactobacillus paracasei* and *Lactobacillus rhamnosus*, had the strongest effect on the yeast [44].

*Lactobacillus reuteri* is a promising bacterium (especially DSM 17938 and ATCC PTA 5289) for its anti-*Candida* properties, confirmed by several studies. In one of these, *Lactobacillus reuteri* was demonstrated to be able to reduce *Candida* load in vivo through co-aggregation, modification of oral pH with production of lactic acid and other organic acids that inhibit the virulence of *Candida* cells, and production of H_2_O_2_ [45].

In a recent in vitro study by Coman et al. (2014), the strains *Lactobacillus rhamnosus* IMC 501 and *lactobacillus paracasei* IMC 502, alone or in combination, showed an inhibitory effect on *Candida* spp. growth [46].

*Lactobacillus delbrueckii ssp. bulgaricus* B1 and *Lactobacillus delbrueckii ssp. bulgaricus* TAB2 were found to fight *Candida*, releasing high amounts of lactic acid [47].

Recently, it was found that *Lactobacillus rhamnosus* GR-1 and *Lactobacillus reuteri* RC-14 modulate *Candida glabrata* virulence, through the complete inhibition of fungal biofilms [48].

In addition, *Lactobacillus acidophilus* ATCC 4356 was found to inhibit the biofilm formation of fungus through in vitro experiments [49]. Biofilm formation is probably reduced through the production of substances called “bacteriocins” by probiotics. Wannun et al. reported the isolation of a bacteriocin, called “fermencin SD11”, from *Lactobacillus fermentum* SD11, a human oral *Lactobacillus*, which has a strong inhibitory effect on oral *Candida* cells [50].

In 1997, Wagner et al. showed that the administration of probiotics could be a prophylactic and therapeutic strategy for mucosal candidiasis [43]. They demonstrated that the presence of four strains of bacteria (*Lactobacillus acidophilus, Lactibacillus reuteri, Lactobacillus casei GG,* and *Bifidobacterium animalis*) in the gastro-intestinal tract of immunodeficient mice reduced the number of *Candida* albicans cells, as well as the incidence and severity of mucosal and systemic candidiasis, prolonging their survival [43].

In a murine model, Matsubara et al. inoculated *Candida albicans* in the oral cavity and subsequently administrated an antifungal drug (nystatin) or probiotics (*Lactobacillus acidophilus* and *Lactobacillus rhamnosus*). At the end of the experiment, colonization by yeast cells was lower in the group that received probiotics (particularly *L. rhamnosus*) than in the group treated with nystatin [51].

In conclusion, even if the mechanism of probiotics’ antifungal effect remains to be fully elucidated, some authors explored it in vitro and in vivo studies, showing that these bacteria may contrast *Candida* spp. infection through different and synergistic mechanisms of action.

In this paper, we performed a systematic review and meta-analysis of clinical studies, randomized controlled trials (RCT), and pre–post intervention studies, with the aim of investigating the efficacy of probiotics (compared with a control treatment or placebo) on oral *Candida* spp. counts in subjects of any age, sex, nationality, or health status.

## 2. Systematic Review and Meta-Analysis 

### 2.1. Materials and Methods

#### 2.1.1. Literature Search

The PRISMA statement guidelines were followed for conducting and reporting a systematic review and meta-analysis [52].

A computerized search of the articles published from inception to 1 February 2019, was conducted in Embase, Medline/PubMed, Cochrane Library central, clinicaltrials.gov databases, and other individual journals sources (Brazilian Dental Journal, Indian Journal of Health Sciences, and Biomedical Research Kleu), using the following search string: (candidosis OR candidiasis OR oral *Candida* spp. OR thrush OR yeast infection) AND (probiotic OR prebiotic OR yogurt OR synbiotic OR *Lactobacillus* OR *Bifidobacteria* OR *Saccaromyces* OR *Bacillus* OR xylitol). In the PubMed database, we activated the filter “Humans”; in Embase, we activated the filter “Research articles”; in Cochrane Library, we activated the filter “Trials”; and, in clinicaltrials.gov, we activated the filter “recruitment: terminated or completed”. No restrictions of language, country, duration of follow-up, and participants’ characteristics (race, age, and sex) were imposed.

#### 2.1.2. Study Selection

Two authors independently reviewed titles and abstracts of the collected articles, applying pre-defined inclusion /exclusion criteria. The inclusion criteria were as follows:

(1) Randomized clinical trials or pre–post intervention studies;
(2) Availability of full text;
(3) Patients regardless of age, race, nationality, sex, and health status;
(4) Comparison between oral intake of probiotics, prebiotics, or synbiotics (of any type and dosage) with a control treatment or a placebo in RCT; and between pre- and post-treatment conditions in pre–post intervention studies;
(5) Outcome measurement expressed in CFU/mL of oral *Candida* spp. counts in saliva or palatal samples.

The exclusion criteria were as follows:

(1) Studies with fewer than 10 participants;
(2) Reviews, articles, and case reports;
(3) Studies with incomplete outcome data;
(4) Studies reporting results in a format which was not suitable for a meta-analysis, for example, without *Candida* spp. counts/carriage.

#### 2.1.3. Data Extraction

The same two authors performed the analysis of the full texts and the data extraction, with the intervention of a third author in the case of poor agreement or discrepancies. Each reviewer independently recorded data in a predefined data extraction form. The following data were obtained from each selected trial: first author name, year of publication, study design, availability of a registered study protocol, setting (institution, city, and country), characteristics of the studied population (mainly age and health status), sample size, number of total participants at the end of follow-up, number of subjects, number of subjects in the treatment and control groups (for RCTs), experimental treatment (strain type or mixture type, dose in CFU/mL, and frequency of administration), control treatment, inclusion and exclusion criteria, follow-up duration, characteristics of the sample, and outcome measure expressed in *Candida* spp. counts (in CFU/mL).

#### 2.1.4. Outcome Assessment 

For each selected study, we calculated the odds ratio of OC (*Candida* spp. counts lower than a threshold of 10^2^, 10^3^, or 10^4^) of treated subjects versus controls, as a measure of treatment effect.

For seven RCTs (Hatakka et al. 2007 [52]; Ishikawa et al. 2015 [53]; Keller et al. 2018 [54]; Kraft-Bodi et al. 2015 [55]; Li et al. 2014 [56]; Myazima et al. 2017 [57]; Petti et al. 2001 [58]), it was possible to calculate the OR and its standard error from the 2 × 2 contingency table of the trial results. For the RCT by Burton et al. 2013 [59], which reported a continuous outcome (mean of *Candida* spp. counts in CFU/mL), we firstly calculated the standardized mean difference (SMD) between the treatment group and the control group; then, we derived the OR and the corresponding standard error according to the Hasselblad and Hedges method [60]. The same approach was also used for two pre–post studies (Rane et al. 2018 [61]; Sutula et al. 2013 [62]). In these cases, we assumed independence between pre and post means, thus overestimating the standard errors.

Rane et al. (2008) [61] reported the results of separated analyses conducted on three different samples of different age (50–59, 60–69, ≥70 years). A fixed-effects meta-analysis was performed on the three results in order to obtain an overall combined estimate and the corresponding standard error.

Miyazima et al. (2017) [57] conducted an RCT with three treatment arms (placebo, treatment 1 with *Lactobacillus acidophilus*, and treatment 2 with *Lactobacillus rhamnosus*). We collapsed the results for the two experimental treatments in order to obtain an overall OR of treatment vs. placebo.

For the pre–post studies by Lopez-Jornet et al. 2018 [63] and Mendonca et al. 2012 [64], which reported the status of the patient in binary form, the McNemar OR was calculated [65].

#### 2.1.5. Risk of Bias

The assessment of quality of the randomized clinical trials was performed using Review Manager 5.3 software, according to the Cochrane Handbook guidelines [66].

The two reviewers expressed, for each of the eight selected RCTs, their independent judgment (low risk, high risk, or unclear) on the following domains: random sequence generation, allocation concealment, blinding of participants and personnel, blinding of outcome assessment, incomplete outcome data, selective reporting, and other bias. 

The assessment of quality of the pre–post intervention studies was performed using the quality assessment tool for pre–post studies with no control group developed by the United States (US) National Heart, Lung, and Blood Institute (NHLBI) [67]. 

In the case of disagreement between the two authors, a third investigator was involved to resolve the controversy.

#### 2.1.6. Statistical Analysis

A Bayesian random-effects meta-analysis model was specified to combine the results of the selected studies and, successively, to combine the results from the subset of the RCTs [68]. Let *b_i_* be the estimate of the log (OR) from the *i*th study, and *s_i_* be the estimate of the corresponding standard error (*i* = 1, 2, …, *n*). The random-effects meta-analysis assumes that the study-specific effects *b_i_* are mutually independent and follow the following model:

*b*_*i*_ = *β* + *u*_*i*_ + *ε*_*i*_,    *u_i_* ~ *N* (0, *τ*^2^),     *ε_i_* ~ *N* (0, *s_i_*^2^), (1)

where *β* is the overall meta-analytic effect, *_i_* is a random effect normally distributed with variance *τ^2^*, and *ε_i_* is an error term with known variance; *u_i_* and *ε_i_* are assumed to be independent. The meta-analysis model accounts for possible heterogeneity among studies through the random terms *u_i_*, and the variance *τ^2^* expresses the heterogeneity among studies. In the Bayesian formulation of the model, we need to specify prior distributions. Two non-informative priors were specified on the hyperparameters *β* and *τ^2^*.

We used MCMC methods to obtain a sample from the joint posterior distribution of the parameters [69]. The posterior distribution of β was summarized in terms of mean and 95% credibility interval (CrI), i.e., the 2.5th and 97.5th percentiles of the posterior distribution. The posterior distribution of the *I^2^* index, which expresses the percentage of total variance captured by *τ^2^*, was summarized in terms of median and 95% CrI.

Sensitivity analyses were performed to evaluate the discrepancy between the results of the meta-analysis on the RCTs and the results obtained excluding the two RCTs that enrolled only subjects wearing dentures and the RCT that enrolled children.

When the meta-analysis included fewer than three studies, a fixed-effects models was adopted where only the within-study component of the variance was accounted for.

The presence of publication bias was evaluated by inspecting a funnel plot [70,71] and calculating the Begg’s test [72]. All analyses were performed with R software (v3.5.1) [73] and the library OpenBUGS (v3.0.7) [74].

### 2.2. Results

#### Study Selection

The initial search identified 2490 articles.

In total, 87 studies were excluded for duplication. Of the remaining 2403 papers, 2385 were excluded after the screening for title and abstracts because they did not fulfil the inclusion criteria.

Among the 18 remained eligible articles, four were eliminated because the full text was not available, the outcome was inappropriate, or the results were not reported. Finally, 12 studies were included in the meta-analysis.

**Figure 1:**
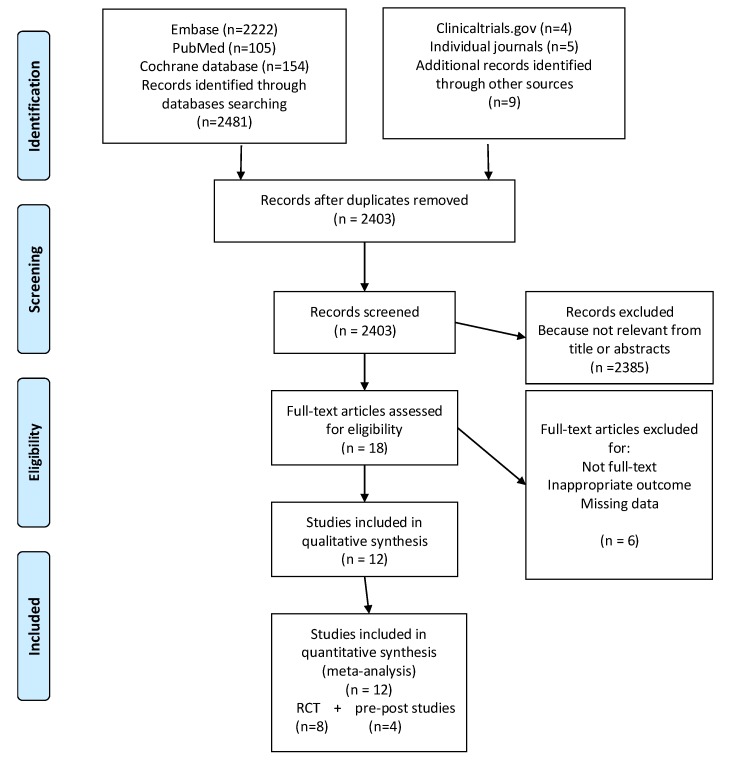
PRISMA flow diagram.

Figure 1 illustrates the selection process, according to the PRISMA statement 2009.

### 2.3. Characteristics of the Included Studies

An overview of the included studies is reported in Table 2, which includes the reference, study design, setting, enrolled population, number of participants, intervention, comparison, follow-up, sample type, and outcome measurement.

The 12 papers included in the analysis were published from 2001 to 2018. Eight studies were RCTs [53,54,55,56,57,58,59,60], and four were pre–post intervention studies [62,63,64,65].

The sample size ranged from 21 to 192. The total number of participants was 843, with an age between five and 100 years, with elderly people (70–100 years) being the most represented (*n* = 395). Three studies included only denture wearers [54,58,62], and one study involved schoolchildren [60].

The patients were from 10 countries: Brazil, China, Denmark, Finland, India, Italy, New Zealand, Sweden, and United Kingdom. Three studies were from Brazil [54,58,65].

The studies investigated a total of 16 strains (alone or in combination) of probiotics. The most represented were *Lactobacillus* spp., followed by *Bifidobacterium* spp., *Saccaromyces* spp., and in one case *Propionibacterium* spp., at doses from 5 × 10^5^ to 5 × 10^9^ CFU/mL one, two, or three times a day or less frequently (such as three times a week or every two weeks). None of the studies included prebiotics or synbiotics.

Most probiotics were given though lozenges or capsules, or with dairy products such as cheese, milk, or yogurt in some studies.

The length of the follow-up ranged from a minimum of four weeks to a maximum of 16 weeks.

The reported outcomes measures were different among studies. Three studies reported the means of CFU/mL, two studies reported the presence (yes/no) of *Candida* spp. carriage, and seven studies reported the *Candida* spp. carriage (yes/no) based on different thresholds of *Candida* spp. counts (10^2^, 10^3^, or 10^4^).

### 2.4. Evaluation of the Risk of Bias for RCTs

**Figure 2:**
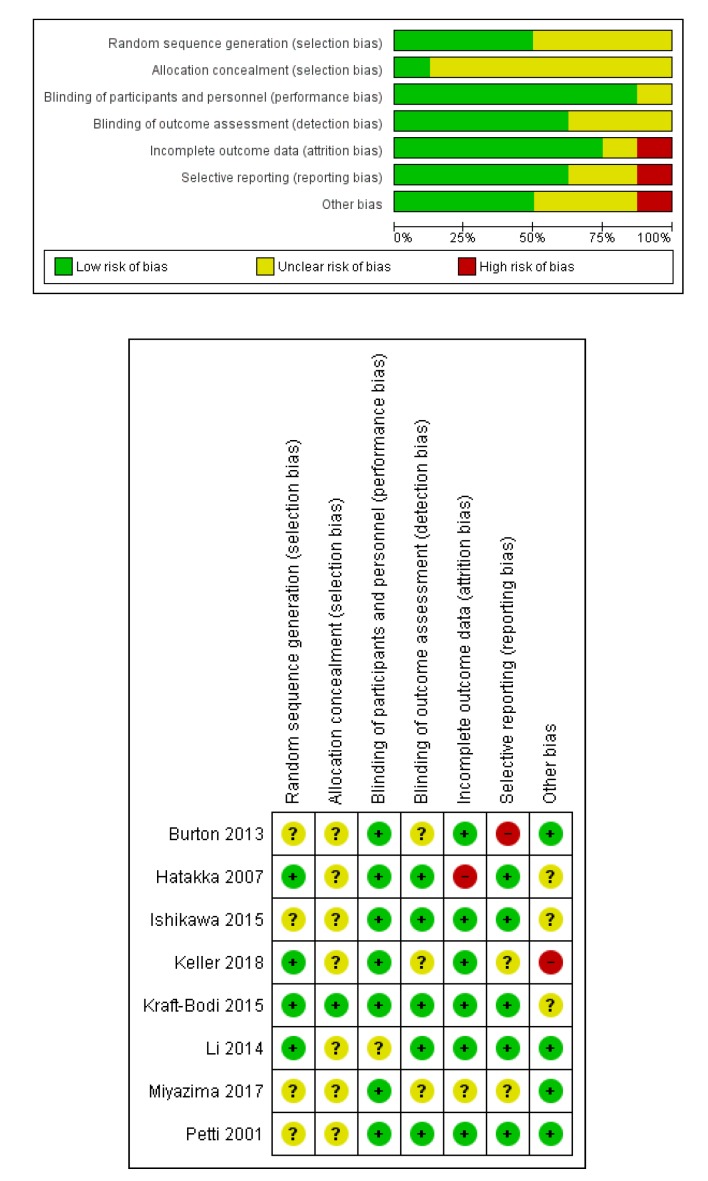
Risk of bias summary and graph. Green: low risk, yellow: unclear risk, red: high risk. In total, 50% of RCTs appropriately described the random sequence generation process, but only one reported the allocation concealment in detail. In most studies (87.5%), participants and personnel were blinded; in five studies, the outcome assessors were also blinded. Six studies out of eight had a low risk of incomplete data, with only one (Hatakka et al. 2007 [53]) being classified as having a high risk because 30% of participants dropped out during the study. Regarding selective reporting, one study (Burton et al., 2003 [60]) was judged to have a high risk because, in the article, the outcome of interest was incompletely reported. However, after e-mail contact with the authors, the reviewers received the data required. Finally, the reviewers assigned high risk to Keller et al., 2018 [55], because, as declared by authors, “fewer participants completed the study as projected, and the study was terminated before completion because of recruitment problems”.

The results of the risk of bias evaluation are reported in Figure 2.

### 2.5. Evaluation of the Risk of Bias for Pre–Post Intervention Studies

**Figure 3:**
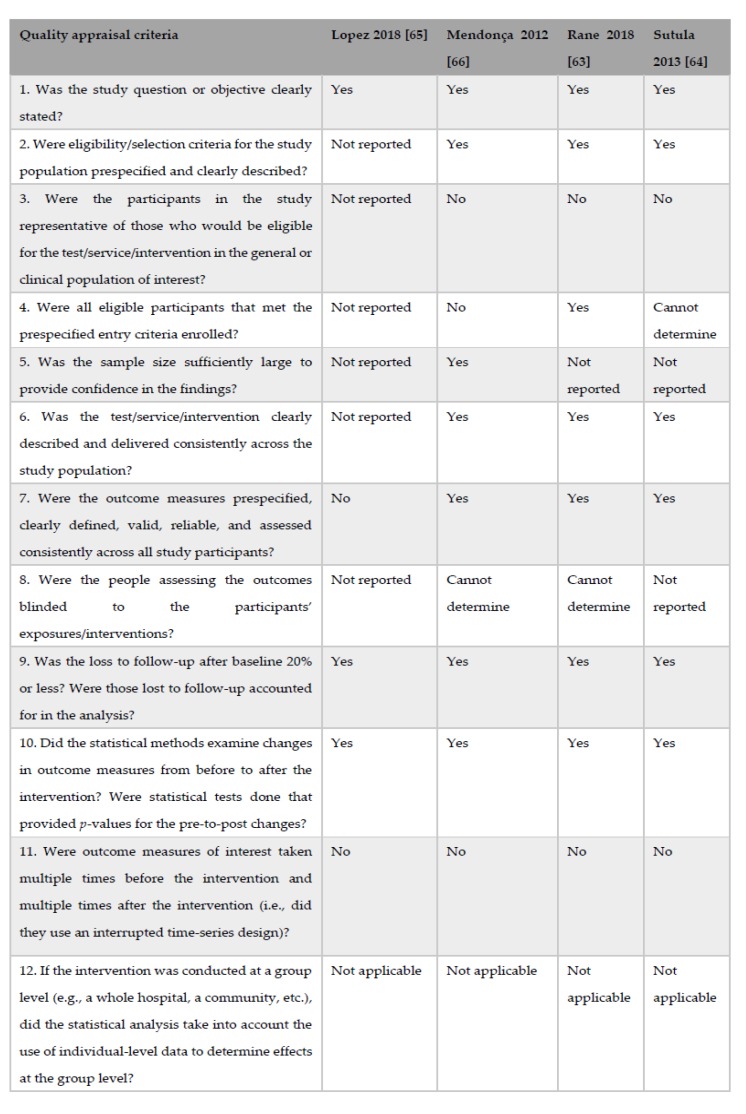
Quality appraisal criteria for pre–post intervention studies.

On the basis of the quality appraisal criteria proposed by the NHLBI [68] for pre–post intervention studies with no control group, the study by Lopez et al. (2018) [64] was classified as having poor quality because the paper consisted of a correspondence letter which provided poor information about the trial. The other three studies were susceptible to some bias, but not sufficient to invalidate the results (Figure 3). Thus, they were assigned to the fair quality category.

### 2.6. Meta-Analysis Results

**Figure 4:**
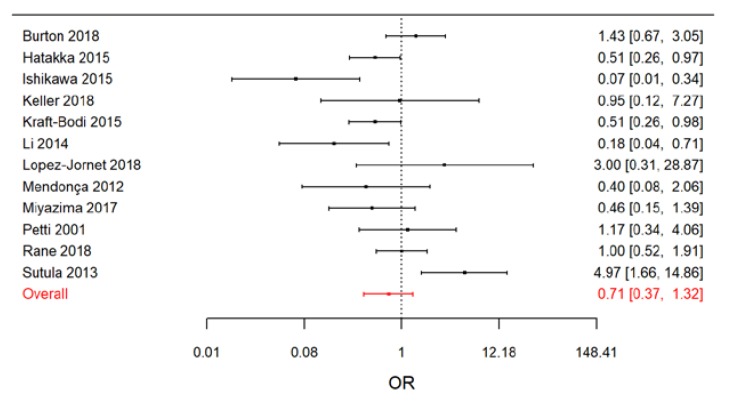
Forest plot from the Bayesian random-effects meta-analysis on all the selected studies.

The results of the Bayesian random-effects meta-analysis conducted on the 12 evaluated studies are reported in the Figure 4.

Overall, we estimated that the OC odds ratio in the treated patients was around 30% lower than in the controls (OR = 0.71; 95% CrI: 0.37, 1.32). However, it should be noted that the 95% confidence interval of the overall OR was not completely included in the beneficial effect region (OR < 1). The *I^2^* index, with a posterior median of 56.3% (95% CrI: 6.0%, 84.4%), suggests the presence of a relevant heterogeneity among studies.

**Figure 5:**
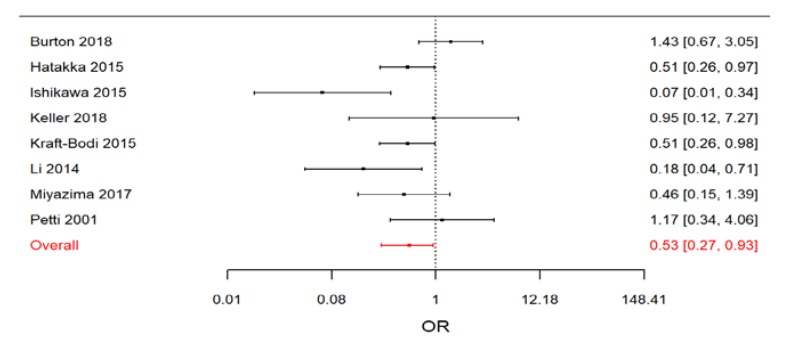
Forest plot from the Bayesian random-effects meta-analysis on randomized controlled studies.

When we restricted the meta-analysis to RCTs, a clear beneficial effect of treatment arose (Figure 5).

The estimated effect size was lower than in the previous analysis (OR = 0.53; 95% CrI: 0.27, 0.93), but the precision of the estimate was larger. As expected, this subset of studies appeared to be less heterogeneous (posterior median of *I^2^* = 32.2%; 95 CrI: 0.3%, 84.0%), even if a relevant discrepancy between results was still present.

**Figure 6:**
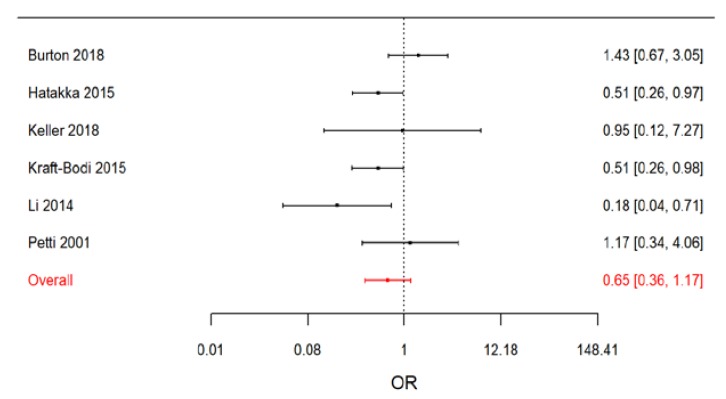
Forest plot from the Bayesian random-effects meta-analysis of the randomized controlled studies on non-denture wearers.

In a sensitivity analysis, we performed a stratified meta-analysis distinguishing between RCTs performed on denture wearers and RCTs on non-denture wearers. The effect of treatment appeared to be larger in denture wearers, with an OR equal to 0.65 (95% CrI: 0.36, 1.17) for non-denture wearers (Figure 6) versus an OR equal to 0.19 (95% CrI: 0.03, 1.29) for denture wearers.

However, in interpreting this result, we should account for the fact that the meta-analysis on denture-wearing patients relied on only two studies. 

**Figure 7:**
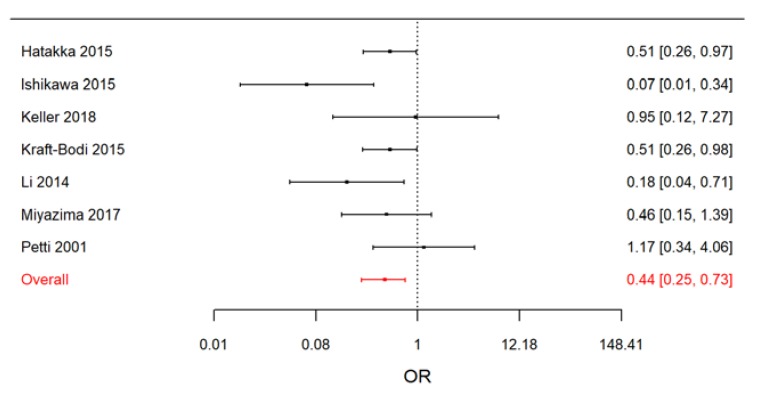
Forest plot from the Bayesian random-effects meta-analysis of the randomized controlled studies on adults.

When, in a second sensitivity analysis, we excluded from the RCTs the study by Burton et al. (2013) [60], which was conducted on schoolchildren, we observed a slight increase in effect size (OR = 0.44; 95% CrI: 0.25, 0.73) and a reduction in *I^2^* (posterior median of *I^2^* = 7.0%; 95% CrI: 0.2%, 76.2%) (Figure 7).

A summary of the meta-analyses results is presented in Table 3.

**Figure 8:**
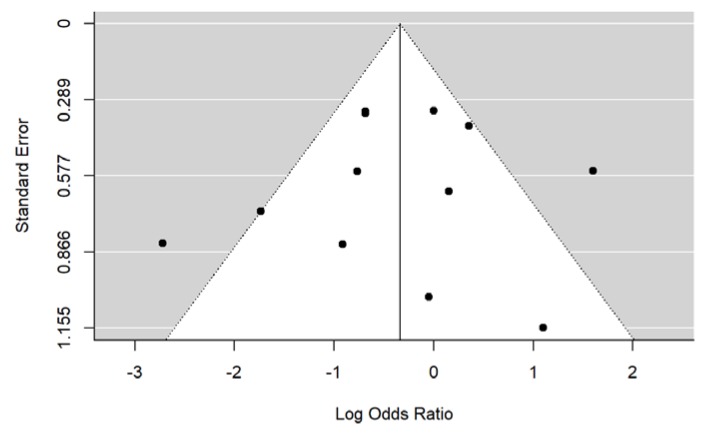
Funnel plot of publication bias.

From the inspection of the funnel plot, no evidence of publication bias arose (Figure 8).

These results were confirmed by the Begg’s test (*p* = 0.80 for the meta-analysis on the RCTs, *p* = 1 for the meta-analysis on the 12 studies).

## 3. Discussion

Currently, fungal infections are widespread, especially in developed countries. A higher incidence of *Candida* spp. infections is associated with some predisposing factors such as the use of dentures, malnutrition, endocrine disorders, smoke, and some chronic diseases such as diabetes, HIV infection, and cancer [75]. The anti-OC treatment is mainly based on antifungal drugs, but different clinical types of OCs and the increasing number of multi-resistance phenotypes of *Candida* spp. represent current threats for public health. Consequently, the development of alternative therapeutic or complementary measures appears necessary to prevent the emergence of fungal resistance [76].

Many studies demonstrated that probiotics represent an efficient alternative treatment against *Candida* spp. infections. Moreover, they are easy to use and, thus, these products are usually well accepted by the patients [77]. The present study provides an overview of the literature on this issue, as well as a quantitative analysis that combines the results of independent studies of different design.

Both the meta-analysis on the 12 selected studies and the meta-analysis conducted on the subset of the RCTs indicated that the treatment had a beneficial effect on reducing oral *Candida* spp. counts. 

As expected, the heterogeneity among studies was relevant because we combined studies of different design, which focused on different populations, used different treatments and doses, and were affected by different kinds and levels of bias. Our sensitivity analyses highlighted that part of the observed heterogeneity could be due to an actual difference of the treatment effect when used in different populations. For example, we found that the effect on denture wearers was larger than the effect estimated on non-denture wearers. The result on denture wearers relied only on two RCTs and should be interpreted with caution, but it is suggestive of a true difference. The larger reduction in the number of *Candida* spp. colonies in these patients could be caused by the direct application of probiotic products on the denture surface [57]. This hypothesis supports the idea that a lower effect of probiotics could be due to the low frequency of usage, number of probiotic cells, and delivery system, which exert an effect on the period of probiotics maintenance at the oral cavity. In this sense, the development of a mucoadhesive buccal drug delivery system [78], in order to enable the prolonged retention at the site of action, could improve the therapeutic outcome. An indication in favor of the relevance of the number of doses per day seems to arise also from the comparisons of the ORs in our meta-analysis. If we focus on RCTs on non-denture wearers, a larger effect was reported in Li et al. (2014) [60], where the treated patients received three doses per day instead of one or two. Three doses were administered also in Keller et al. 2018 [55], but this study was affected by recruitment problems, and the result relied on a very small number of subjects.

A second relevant source of heterogeneity was related to the fact that different microbial probiotic strains could have different effects on the reduction of *Candida* spp. *c*ounts [58]. For example, Matsubara and colleagues found that, in a mice model, the treatment with *Lactobacillus rhamnosus* Lr-32 was more effective than the treatment with *Lactobacillus acidophilus* on the *Candida* spp. colonization levels [51]. Unfortunately, as the number of studies collected from the literature was too small to build a network of comparisons involving multiple treatments, we considered all treatments as having the same effect, which was clearly a very strong assumption.

Therefore, summarizing the obtained results, we can conclude that probiotics have a protective role in the *Candida s*pp. infection and especially colonization. As previously reported, the anti-*Candida* properties can be explained in different ways, such as (a) through co-aggregation, modification of oral pH, and production of H_2_O_2_ [45], (b) through releasing high amounts of lactic acid [47], and (c) through the complete inhibition of fungal biofilms [48,49]. However, these positive effects are highly linked to the administration method, the dosage, and the used probiotics strains. In addition, we did not find studies on prebiotics and synbiotics eligible for our meta-analysis. The effect of these products on the oral candidiasis must be better investigated in order to discover novel antifungal effects. In fact, some studies demonstrated that the combination of probiotics and prebiotics (synbiotics) can be very effective in infections [79,80].

Our results suggest planning a new clinical study to evaluate the real effectiveness of probiotics treatment in *Candida* spp. infection. The focal points of the study should be (1) the age stratification of the patients (old or adult), (2) the administration method (topic or oral), the type (lozenges or capsules), the dosage, and the treatment duration, (3) the choice of appropriate probiotic strains (*Lactobacillus* spp., *Bifidobacterium* spp., *Saccaromyces* spp., or *Propionibacterium* spp.), and (4) the length of the patient follow-up.

Our study also had other limitations. Firstly, the number of studies included in the meta-analysis was small, in particular when we focused on the RCTs. Secondly, some of the studies had a high risk of bias. Thirdly, with the aim of providing an overview of the literature, we did not apply strong exclusion criteria, at the price of a larger heterogeneity among studies. For the same reason, we sometimes had to adopt approximations to obtain a common comparable effect measure (OR) from the results reported in the original papers; this could have introduced a certain degree of bias in the meta-analysis.

## 4. Conclusions

In conclusion, our meta-analysis is one of the first that critically evaluated the impact of probiotics in oral candidiasis and, on the basis of the meta-analysis results, despite the high heterogeneity among studies, we are confident in declaring that the treatment can have a beneficial effect on reducing oral *Candida* spp. counts.

## Figures and Tables

**Table 1:** Factors related to oral candidiasis (OC).

Factors Related to Oral Candidiasis
Iatrogenic factors
Antineoplastic agents [12]
Broad-spectrum antibiotics [13]
Inhaled corticosteroids [14]
Substance abuse [15,16]
Health conditions
Anemia [17]
Immunosuppression status [18]
Nutritional deficiencies [13]
Xerostomia [19]
Diseases
Cancer [20]
Cushing syndrome [13]
Diabetes mellitus [21,22]
Human immunodeficiency virus (HIV) [23]
Other factors
Age [17]
Denture wearing [20]
Pregnancy [24]
Smoke [16]

**Table 2:** Overview of the included studies. RCT—randomized controlled trial; CFU—colony-forming unit; OR—odds ratio; CI—confidence interval.

Reference	Study Design	Setting	Studied Population	No. of Participants	Intervention	Comparison	Follow-up	Sample Type	Outcome
Burton et al. 2013 [60]	RCT	Schools with dental clinics City: Dunedin Country: New Zealand	Child population, schoolchildren with active caries. Age: 5 to 10 years (mean 8.5 years)	Total: 83; 40 in the probiotic group, 43 in the placebo group	Two lozenges with *S. salivarius* (each lozenge 3.6 × 10^9^ CFU of strain), two times a day, one in the morning and one at night, for three months	Placebo. Lozenges with identical appearance and taste, without probiotics	3 months	Saliva samples	OR* 1.427 95% CI (0.667–3.054)
Hatakka et al. 2007 [53]	RCT	Homes and sheltered housing units City: Helsinki Country: Finland	Elderly people, aged 70–100 years	Total: 192; 92 in the probiotic group, 100 in the placebo group.	Daily 50 g of Emmental-type probiotic cheese divided into two portions, with *Lactococcus lactis* and *Lactobacillus helveticus* as starter cultures and 10^7^ CFU/g of each probiotic strain: *L. rhamnosus GG* (ATCC 53103), *L. rhamnosus LC705*, and *Propionibacterium freudenreichii* ssp. *shermanii JS*	Daily 50 g of edam type cheese, divided into two portions, with *Lactococcus lactis* as starter culture without the addition of other probiotic strains	16 weeks	Saliva samples	OR 0.505 95% CI (0.263–0.970)OR calculated for a cut-off of *Candida* ≥10^4^ CFU/ml
Ishikawa et al. 2015 [54]	RCT	Patients seeking dental treatment (complete denture) at the School of Dentistry, University of São Paulo City: São Paulo Country: Brazil	Denture wearers harboring *Candida* spp. in the oral cavity with no clinical symptoms, aged (mean) 61.8 ± 8.5 years	Total: 55. 30 in the probiotic group. 25 in the placebo group	1 capsule/day containing lyophilized cultures (obtainedfrom HardiStrain® – Probiotics) *of L. rhamnosus* HS111, *L. acidophilus* HS101, and *Bifidobacterium bifidum* combined in equal amounts, reaching 108 CFU (3.3 × 10^7^ CFU of each) per capsule	Placebo 1 capsule/day with same characteristics as the probiotic product, but without the probiotic bacteria	5 weeks	Palatal mucosal samples	OR 0.066 95% CI (0.013–0.338)OR calculated for a cut-off of *Candida* ≥10^4^ CFU/ml
Keller et al. 2018 [55]	RCT	Clinic for oral medicine City: Copenhagen Country: Denmark	Patients attending the Clinic for Oral Medicine, aged median (67) years, with diagnosis of oral lichen planus	Total: 22.9 in the probiotic group, 13 in the placebo group	Pre-treatment: all patients were treated with the current conventional treatment regimens at the Clinic for Oral Medicine, including those who required additional conventional treatment during the 1-year study period Patients diagnosed with oral candidiasis were treated withnystatin, patients without oral candidiasis were treated with steroid, fluocinolone acetonide gel 0.025% Treatment: probiotic lozenges containing two strains of the probiotic bacteria *L. reuteri* (DSM 17938 and ATCC PTA 5289) dissolved intra-orally three times daily (morning, noon, and evening just before bedtime) for 16 weeks	Pre-treatment: the same of intervention group Treatment: placebo lozenges without probiotic bacteria	16 weeks	Saliva samples	OR 0.952 95% CI (0.125–7.275)OR calculated with cut-off *Candida* carriage yes/no
Kraft- Bodi et al. 2015 [56]	RCT	Nursing homes. Country: south of Sweden	Elderly people, aged (mean) 88 years	Total: 174; 84 in the probiotic group, 90 in the placebo group	2 lozenges daily the morning and in the early evening, containing a minimum of 10^8^ live bacteria of each strain of the probiotic bacterium *Lactobacillus reuteri* (DSM 17938 and ATCC PTA 5289; Prodentis™, Biogaia® AB, Lund, Sweden)	Placebo lozenges without active bacteria	12 weeks	Saliva samples	OR 0.505 95% CI (0.259–0.984)OR calculated for a cut-off of Candida ≥ 10^4^ CFU/ml
Li et al. 2014 [57]	RCT	Department of Oral Medicine, West China College of Stomatology, Sichuan University City: Sichuan Country: China	Patients with clinically and microbiologically proven *Candida*-associated stomatitis (detection rate of *Candida albicans* in the saliva >10^2^ CFU mL^−1^), aged (mean, SD) 64 ± 10.75 years	Total: 65; 34 in the probiotic group, 31 in the control group	Pre-treatment: administration orally of 2% sodium bicarbonate solution and then application of 2% nystatin pasteTreatment: four lozenges containing the mixture of *B. longum* (5 × 10^6^ CFU in 0.5 g of skim milk powder per tablet), *L. bulgaricus* (5 × 10^5^ CFU in 0.5 g of skim milk powder per tablet), and *S. thermophilus* (5 × 10^5^ CFU in 0.5 g of skim milk powder per tablet)The medication was applied three times daily for 4 weeks.	2% sodium bicarbonate solution and 2% nystatin paste	4 weeks	Saliva samples	OR 0.176 95% CI (0.044–0.710)OR calculated for a cut-off of *Candida* ≥10^2^ CFU/mL
Lopez-Jornet et al. 2018 [64]	Before–after study	Clınica Odontologica Universitaria Hospital Morales MeseguerCity: Murcia. Country: Spain	Patients, aged (mean) 71.2 years	Total: 27	*Lactobacillus reuteri* DSM 17938 (German Culture Collection of Microorganisms) and ATCC PTA 5289 (American Type Culture Collection) (GUM Periobalance®, Sunstar) one tablet per day for 28 days		28 days	Saliva samples	OR 3.00095 %CI (0.312–28.842)OR calculated with a cut-off of *Candida* >10^2^ CFU/mL
Mendonça et al. 2012 [65]	Before–after study	City: Taubaté Country: Brazil	Healthy women aged 65 or older who lived in the city of Taubaté, SP, Brazil	Total: 42	1 g (content of 1 envelope) of the probiotic Yakult LB® (*Lactobacillus casei* and *Bifidobacterium breve*, 2 × 10^7^ to 10^9^ and 5 × 10^7^ to 10^9^ CFU/mL, respectively), 3 times a week, at the same hour, for 30 days		30 days	Saliva samples	OR 0.400 95% CI (0.078–2.062)OR calculated with a cut-off *Candida* carriage yes/no
Miyazima et al. 2017 [58]	RCT	School of Dentistry, University of São Paulo City: São Paulo Country: Brazil	Denture-wearing patients seeking for dental treatment (complete denture), aged (mean, SD) 64.4 ±12.07 years	Total: 60; 20 in each group (treatment 1, treatment 2, control)	Treatment 1, T1 group: fresh cheese added with probiotics containing 8 to 9 log CFU∙g^−1^ *of L. acidophilus* NCFMTreatment 2, T2 group: fresh cheese added with probiotics containing 8 to 9 log CFU∙g^−1^ of *L. rhamnosus* Lr-32	PlaceboControl group (C group): fresh cheese with no added probiotics	8 weeks	Mouth-rinse samples	OR 0.464 95% CI (0.155–1.392)OR calculated for a cut-off of *Candida* ≥10^3^ CFU/mL
Petti et al. 2001 [59]	RCT	Country: Italy	Adult volunteers, aged (mean) 28.2 years	Total: 42; 20 in the yoghurt group, 22 in the control group	125 g of fruit yoghurt twice daily, between breakfast and lunch, and between lunch and dinnerThen, 2 weeks without yogurt intake	125 g of fruit soybean ice cream twice daily, between breakfast and lunch, and between lunch and dinner Then, 2 weeks without soybean ice cream intake	16 weeks	Saliva samples	OR 1.167 95% CI (0.335–4.060)OR calculated for a cut-off of *Candida* between 3.5 and 7.6 × 10^2^ CFU/mL
Rane et al. 2018 [62]	Before–after study	Country: India	Healthy complete denture wearers, aged ≥50 years	Total: 60; 20 in group A (age 50–59 years), 20 in group B (age 60–69 years), 20 in group C (age ≥ 70 years)	Once daily, 1 capsule content of probiotic (Probiotic immune®, Zenith nutrition) in the palatal region of the cleaned maxillary denture		5 weeks	Palatal mucosa samples	OR* group A 0.891 95% CI (0.290–2.736)OR group B 1.323 95% CI (0.431–4.063)OR group C 0.846 95% CI (0.276–2.598)
Sutula et al. 2013 [63]	Before–after study	Manchester Metropolitan University City: Manchester. Country: United Kingdom	Healthy dentate volunteers, aged (mean, SD) 32 ± 11.5 years	Total: 21	One bottle per day of drink milk Yakult ®, containing a minimum of 6.5 × 10^9^ viable cells of probiotic *L. casei* strain Shirota, for 4 weeks		4 weeks	Saliva samples	OR* 4.967 95% CI (1.662–14.843)

* OR derived from the standardized mean difference (SMD) between the treatment and the control group, according to the Hasselblad and Hedges method [60].

**Table 3:** Summary of the results from the Bayesian random-effect meta-analyses. CrI—credibility interval.

Type of Meta-Analysis	Meta-Analytic Estimate (OR) (95% CrI)	*I*^2^ (95% CrI)
All 12 studies	0.71 (0.37–1.32)	56.3 (6.0–84.4)
Only RCTs	0.53 (0.27–0.93)	32.2 (0.3–84.0)
RCTs with non-denture wearers	0.65 (0.36–1.17)	17.6 (0.3–81.8)
RCTs with denture wearers ^1^	0.19 (0.03–1.29)	
RCTs with adult patients	0.44 (0.25–0.73)	7.0 (0.2–76.2)

^1^ Results from the fixed-effects model.

## PRISMA 2009 Checklist statement

The authors read the PRISMA 2009 Checklist, and the manuscript was prepared and revised according to the PRISMA 2009 Checklist.
